# Genetic regulation of antibody responsiveness to immunization in substrains of BALB/c mice

**DOI:** 10.1111/imcb.12199

**Published:** 2018-10-14

**Authors:** Hazel C Poyntz, Angela Jones, Ruy Jauregui, Wayne Young, Aurélie Gestin, Anna Mooney, Olivier Lamiable, Eric Altermann, Alfonso Schmidt, Olivier Gasser, Laura Weyrich, Christopher J Jolly, Michelle A Linterman, Graham Le Gros, Edwin D Hawkins, Elizabeth Forbes‐Blom

**Affiliations:** ^1^ Malaghan Institute of Medical Research Victoria University of Wellington Gate 7, Kelburn Parade Wellington 6012 New Zealand; ^2^ High‐Value Nutrition National Science Challenge New Zealand; ^3^ Grasslands Research Centre AgResearch Tennent Drive Palmerston North New Zealand; ^4^ Riddet Institute Massey University Palmerston North 4474 New Zealand; ^5^ Australian Centre for Ancient DNA University of Adelaide North Terrace Adelaide SA 5005 Australia; ^6^ Centenary Institute and Sydney Medical School University of Sydney Missenden Road Sydney NSW 2050 Australia; ^7^ Lymphocyte Signaling and Development Babraham Institute Cambridge CB22 3AT UK; ^8^ Walter and Eliza Hall Institute of Medical Research Parkville VIC 3052 Australia; ^9^ Department of Medical Biology University of Melbourne Parkville VIC 3010 Australia; ^10^ Present address: Nestlé Institute of Health Sciences Nestlé Research Lausanne Switzerland

**Keywords:** Class‐switch recombination, gene regulation in immune cells, humoral immunity, vaccines

## Abstract

Antibody‐mediated immunity is highly protective against disease. The majority of current vaccines confer protection through humoral immunity, but there is high variability in responsiveness across populations. Identifying immune mechanisms that mediate low antibody responsiveness may provide potential strategies to boost vaccine efficacy. Here, we report diverse antibody responsiveness to unadjuvanted as well as adjuvanted immunization in substrains of BALB/c mice, resulting in high and low antibody response phenotypes. Furthermore, these antibody phenotypes were not affected by changes in environmental factors such as the gut microbiota composition. Antigen‐specific B cells following immunization had a marked difference in capability to class switch, resulting in perturbed IgG isotype antibody production. *In vitro,* a B‐cell intrinsic defect in the regulation of class‐switch recombination was identified in mice with low IgG antibody production. Whole genome sequencing identified polymorphisms associated with the magnitude of antibody produced, and we propose candidate genes that may regulate isotype class‐switching capability. This study highlights that mice sourced from different vendors can have significantly altered humoral immune response profiles, and provides a resource to interrogate genetic regulators of antibody responsiveness. Together these results further our understanding of immune heterogeneity and suggest additional research on the genetic influences of adjuvanted vaccine strategies is warranted for enhancing vaccine efficacy.

## Introduction

Antibody‐mediated immunity provides both short‐term protection from pathogens and long‐lived immunological memory for the lifetime of an organism. The majority of current vaccines confer protection by stimulating the production of threshold titers of IgG isotype antibodies.^1^ However, there is a high degree of heterogeneity in the antibody response across the human population, with a proportion of individuals failing to meet protective thresholds of antibody titers.^1^ Previous investigations have established genetic contributors to antibody responsiveness,^2^, ^3^ and emerging data illustrates that environmental factors including the gut microbiota regulate antibody heterogeneity following immunization.^4^, ^5^, ^6^, ^7^, ^8^

Antibody production can be elicited via T‐dependent and ‐independent responses, through cognate antigen binding to the B‐cell receptor.^9^, ^10^, ^11^, ^12^, ^13^, ^14^ In a T‐dependent response, activated B cells can differentiate to germinal center (GC) B cells with provision of signals from pre‐T follicular helper (Tfh) cells and form a germinal center structure within the lymphoid tissue.^15^, ^16^ Alternatively, activated B cells may form an extra follicular antibody response, a fate shared with B cells eliciting response to T‐independent antigen.^17^, ^18^, ^19^ Provision of signals, such as CD40 and cytokine, induce class‐switch recombination (CSR) within activated B cells.^20^, ^21^, ^22^ This switch mechanism alters the immunoglobulin (Ig) isotype encoded by the B cell through a unique process of intrachromosomal deletion, and is critical for the protective antibody response because of the Ig constant region dictating the effector function of secreted antibodies.^23^, ^24^ The resulting class‐switched plasma cells and memory B cells are key to meeting protective thresholds of antibody titers.^25^, ^26^, ^27^ Dysfunction in any of these stages may lead to a low response phenotype.^28^, ^29^ A significant understanding of the mechanisms that regulate antibody production has been provided by this body of research, yet antibody response heterogeneity remains an issue for vaccinology, indicating a gap in our current knowledge of these mechanisms. Thus, characterizing mediators of antibody responsiveness and relative contribution toward response magnitude will determine factors contributing to population heterogeneity and much needed strategies to boost vaccine efficacy.

We observed that BALB/c substrains from different commercial suppliers (herein referred to as BALB/c A and BALB/c B) had diverse antibody profiles in response to seasonal trivalent influenza vaccine as well as immunization with NP‐OVA adjuvanted with incomplete Freund's adjuvant (IFA). The examination of the stages of antibody generation following immunization demonstrated Tfh cells and class switching (C‐S) within GC B cells were severely perturbed in low antibody responder BALB/c A mice. We established that this C‐S defect was B‐cell intrinsic, shared across CD40 and TLR stimulation pathways and was specific to CSR. Whole genome sequence analysis elucidated polymorphisms in known CSR genes, but a lack of a predicted effect of these polymorphisms on protein activity suggest a role for new or unappreciated regulators of antibody responsiveness in low‐responder BALB/c A mice. These mice and genome sequence data provide a resource to potentially identify new candidates for the development of immune‐adjuvant tools to boost antibody titers to threshold levels required for vaccine efficacy.

## Results

### Divergent antibody responses in substrains of BALB/c mice

**Figure 1:**
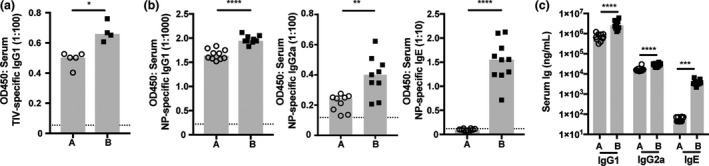
Antibody responsiveness of BALB/c mice from different sources. **(a)** BALB/c A and BALB/c B mice were vaccinated subcutaneously with trivalent influenza vaccine and trivalent influenza vaccine‐specific IgG1 titers were measured 28 days after immunization by ELISA. BALB/c A and BALB/c B mice were vaccinated subcutaneously with NP‐OVA + IFA and **(b)** NP‐specific IgG1, IgG2a and IgE and **(c)** total IgG1, IgG2a and IgE serum titers measured 14 days after immunization by ELISA. Data points represent individual mice and heights of the bar represent the median. Dashed lines represent lower limit of sensitivity, set at blank OD. *P ≤ 0.05, **P ≤ 0.01, ***P ≤ 0.001 and ****P ≤ 0.0001. Data are representative of at least two experiments.

There is mounting evidence that the substrains of mice from different sources have profoundly distinct immune responses, and these immune phenotypes may be influenced by both genetic and environmental factors.^30^, ^31^, ^32^, ^33^, ^34^, ^35^ We sought to examine vaccine‐induced antibody responsiveness in this context with BALB/c mice bred in our facility, but originally obtained from different suppliers. Following immunization with trivalent influenza vaccine, BALB/c A had a significantly attenuated trivalent influenza vaccine‐specific IgG1 response as compared to BALB/c B (Figure 1a). We next investigated the response to NP‐OVA in IFA, and BALB/c A mice produced significantly lower NP‐specific and total IgG1, IgG2a and IgE antibodies following adjuvanted immunization (Figure 1b, c). These data establish that the substrains of BALB/c mice exhibit low‐ and high‐titer antibody responses, with BALB/c A mice possessing a low‐responder phenotype to both unadjuvanted and adjuvanted immunization strategies. We next investigated whether antinuclear antigen‐specific antibody titers were elevated in 24‐week‐old mice, as a surrogate marker of potential autoimmune responses. Antinuclear antigen‐specific antibody levels in both substrains of BALB/c mice were below the reactive serum threshold of the assay (>50 U mL^−1^), suggesting the high response in BALB/c B mice was not linked to an autoimmune phenotype (data not shown).

### Antibody responses are not affected by manipulation of the microbiota in adulthood

**Figure 2:**
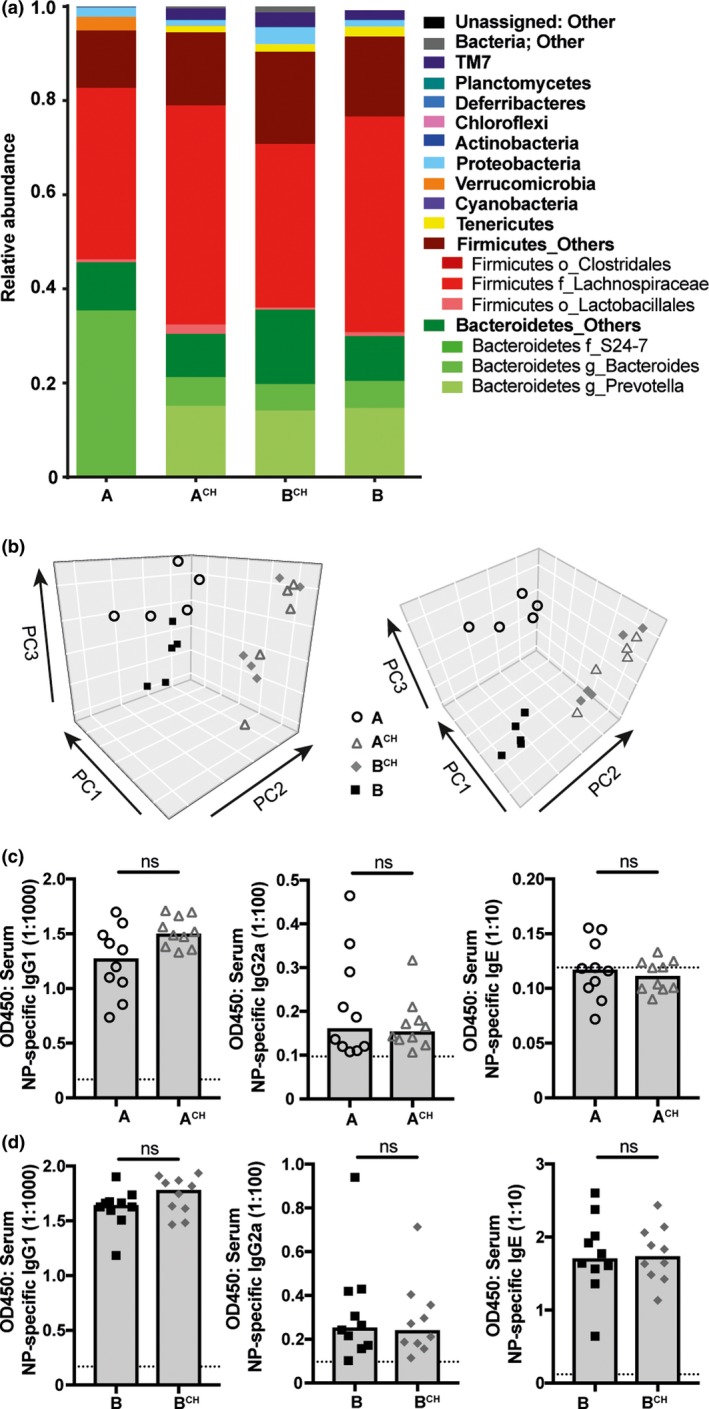
Microbiota composition does not regulate altered antibody responsiveness in BALB/c A and BALB/c B mice. BALB/c A and BALB/c B mice were cohoused to promote microbiota transfer and 4 weeks later vaccinated with NP‐OVA in IFA; cohoused BALB/c A (A^CH^), BALB/c B (B^CH^), BALB/c A and BALB/c B. Gut microbiota composition was determined with taxonomic profiling of fecal bacterial communities from 16s rRNA sequencing. **(a)** Relative abundance at the phyla level with key populations‐of‐interest highlighted at the order, family or genus level and **(b)** principal component analysis of fecal microbiota composition; left and right plot provide two views of the taxonomic diversity. Samples taken 1 day prior to immunization (*n* = 5 per group). NP‐specific IgG1, IgG2a and IgE titers in serum of **(c)** A^CH^ and BALB/c A and NP‐specific IgG1, IgG2a and IgE titers in serum of **(d)** B^CH^ and BALB/c B 14 days after immunization with NP‐OVA in IFA. Data points represent individual mice and heights of the bar represent the median. Dashed lines represent lower limit of sensitivity, set at blank OD. Data are representative of at least two experiments.

To evaluate the variability in gut microbiota between the substrains of BALB/c mice, we employed 16S ribosomal RNA sequencing. The fecal gut microbiota composition observed was substantially different between the BALB/c A and BALB/c B substrains (Figure 2a). However, the level of richness and diversity of the microbial communities were comparable (data not shown). To determine the influence of microbiota composition on antibody response phenotype, a substantial alteration in microbial composition was induced using the established method of cohousing.^34^, ^36^ Cohousing resulted in a marked shift in gut microbial composition in both BALB/c A and BALB/c B cohoused mice (BALB/c A^CH^ and BALB/c B^CH^, respectively), demonstrating cohousing resulted in horizontal microbial transfer (Figure 2a, b). Despite the similar taxonomic profile in A^CH^ and B^CH^ after cohousing, the function of these microbiomes may still be disparate.^37^ To investigate the microbiota influence on antibody response capability, A^CH^ is compared to BALB/c A, and B^CH^ to BALB/c B, as a marked shift from the same starting composition has been induced. In response to immunization with NP‐OVA in IFA, cohousing was not sufficient to alter NP‐specific IgG1, IgG2a and IgE antibody responses in BALB/c A^CH^ as compared to BALB/c A mice (Figure 2c), or BALB/c B^CH^ antibody responses compared to BALB/c B mice (Figure 2d). Moreover, housing mice in the same cage can control for other environmental variables in addition to microbial transfer, thus allowing us to eliminate the influence of other environmental variables on the antibody response. As environmental effects did not influence immunization‐induced antibody production, these data suggest the antibody response capability is genetically regulated in both substrains of BALB/c mice.

### Altered capability of B cells to undergo isotype class switch in low‐responder BALB/c A mice

**Figure 3:**
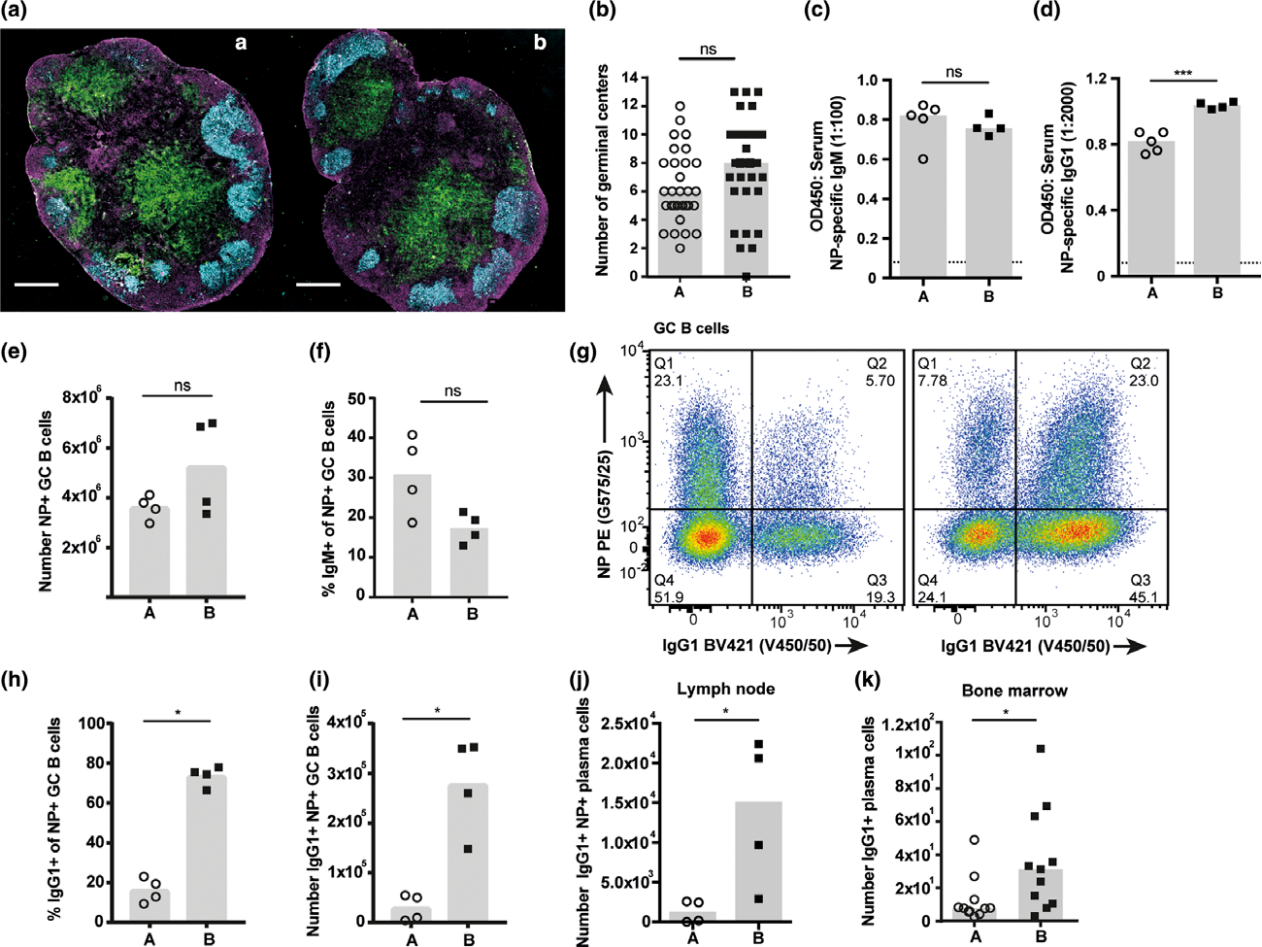
Germinal center B cells from low‐responder BALB/c A mice possess a reduced class‐switch capability. BALB/c A and BALB/c B mice were vaccinated subcutaneously with NP‐OVA + IFA. Draining lymph nodes and serum were taken 14 days after immunization. **(a)** Formation of GC structures was visualized by immunofluorescence imaging; Cyan: GL7, Green: CD4, Magenta: B220. Representative images of one mouse per group. Scale bar 500 μm. **(b)** The number of GL7+ cell clusters within the B‐cell follicle counted per section. **(c)** NP‐specific IgM and **(d)** IgG1 serum titers measured 14 days after immunization by ELISA. **(e)** Number of NP+ GC B cells in the lymph node. **(f)** Frequency of NP+ IgM+ GC B cells in the lymph node. **(g)** Dot plots illustrate IgG1 expression and NP‐specificity within the GC B‐cell population; pre‐gated on size, viability CD138– B220+ GL7+ CD38–. Representative dot plots of one mouse per group. **(h)** Frequency and **(i)** number of NP+ IgG1+ GC B cells in the lymph node. **(j)** Number of NP+ IgG1+ plasma cells in the lymph node; gated on size, viability CD138+ B220–. **(k)** Number of IgG1+ plasma cells in the bone marrow 28 days after immunization with OVA adjuvanted with alum; gated on size, viability CD138+ B220–. Data points represent individual mice and heights of the bar represent the median. Data are representative of at least three experiments.

To determine the mechanisms resulting in the diverse antibody responsiveness, we initially assessed B‐cell frequencies in multiple tissues under homeostatic conditions. B‐cell frequencies in spleen, bone marrow and lymphoid tissues, and frequencies of B‐1, marginal zone and follicular B‐cell subsets within the spleen were all comparable between the substrains of BALB/c mice, suggesting no defects in hematopoiesis or B‐cell homeostasis in low‐responder BALB/c A mice (Supplementary figure 1). We then tracked the formation of the T‐dependent B‐cell response after immunization with NP‐OVA in IFA. GC structure in the draining lymph nodes was comparable between substrains (Figure 3a), and a similar frequency of GC structures were observed (Figure 3b). Serum titers of NP‐specific IgM were also comparable; however, NP‐specific IgG1 titers were significantly reduced in BALB/c A mice (Figure 3c, d). The development of the NP‐specific B‐cell response was also tracked using flow cytometry. No difference in the numbers of NP‐specific GC B cells induced by BALB/c A and B mice was observed, correlating with comparable formation of GC structure observed by imaging (Figure 3e). Of these NP‐specific GC B cells, a similar frequency of IgM isotype was also observed (Figure 3f). However, a reduced frequency of NP‐specific GC cells were of class‐switched IgG1 isotype in BALB/c A, meaning numbers of IgG1^+^ NP‐specific GC B cells were significantly reduced in low‐responder BALB/c A mice (Figure 3g–i). Similarly, the generation of IgG2a^+^ GC B cells in BALB/c A was also reduced (Supplementary figure 2a, b). Taken together, these data indicate BALB/c A mice develop NP‐specific GC B cells and mount an antigen‐specific IgM antibody response comparable to BALB/c B mice. Conversely, the fate of isotype switched responding B‐cell populations is significantly skewed, leading to reduced NP‐specific IgG1, IgG2a and IgE isotypes in BALB/c A mice.

Fewer NP‐specific plasma cells were detected in the draining lymph nodes of BALB/c A mice (Supplementary figure 2c). Additionally, a higher proportion of the NP‐specific plasma cells were IgG1 isotype in BALB/c B as compared to BALB/c A, whereas BALB/c A possessed a higher proportion of IgM isotype as compared to BALB/c B (Supplementary figure 2d, e). This resulted in significantly fewer IgG1^+^ NP‐specific plasma cells in the draining lymph nodes of BALB/c A (Figure 3j). Long‐lived plasma cells migrate to the bone marrow soon after differentiation and so the frequency of C‐S plasma cells was assessed in this compartment.^20^ A significantly reduced number of IgG1^+^ plasma cells were observed in low‐responder BALB/c A mice (Figure 3k). Affinity maturation is a fundamental process within the GC to select high affinity B cells,^38^ and these high affinity B cells receive enhanced Tfh‐derived C‐S signals.^39^ Therefore, reduced C‐S in BALB/c A may be a result of perturbed affinity maturation. We examined the affinity of NP‐specific antibodies as a readout of affinity maturation efficiency; a small but significant difference in NP‐specific IgG1 affinity was observed between the substrains (Supplementary figure 2f). These results demonstrate that the reduced capability of low‐responder BALB/c A GC B cells to undergo productive C‐S leads to reduced antigen‐specific IgG plasma cells forming from the GC response, which in turn infers the altered magnitude of the immunization‐induced class‐switched antibody response.

### Altered capability to mount an appropriate Tfh‐cell response in low‐responder BALB/c A mice

**Figure 4:**
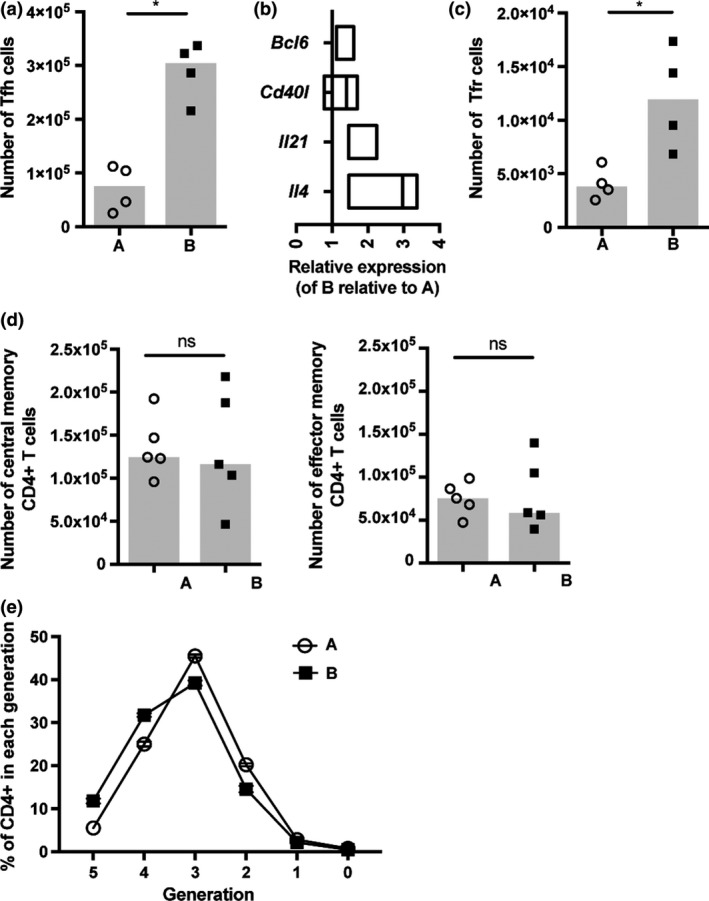
Tfh response capability is altered in low‐responder BALB/c A. **(a)** The number of Tfh cells within draining lymph nodes 14 days after NP‐OVA + IFA immunization was determined by flow cytometry. Tfh gated on size, viability CD3^+^ CD4^+^ CXCR5^+^ PD‐1^+^ FOXP3^–^. **(b)** Tfh cells were sorted from draining lymph nodes 7 days after immunization and expression of *Bcl6*,* Cd40l*,* Il21* and *Il4* determined by qRT‐PCR. The relative expression of each target to 18S is presented, calculated relative to BALB/c A responsiveness that is set at 1. **(c)** Number of Tfr cells and **(d)** central memory and effector memory CD4^+^ T cells within draining lymph nodes 14 days after NP‐OVA + IFA immunization was determined by flow cytometry. CD4^+^ T cells gated on size, viability CD3^+^ CD4^+^; Tfr: CXCR5^+^ PD‐1^+^ FOXP3^+^, central memory: CD62L^+^ CD127^+^ CD44^+^ and effector memory: CD62L^–^ CD127^+^ CD44^+^. **(e)** *In vitro* proliferation of naïve CD4^+^ T cells in response to αCD3, αCD28 and IL‐2 stimulation. Data points represent individual mice and heights of the bar represent the median. Data are representative of at least three experiments.

Tfh are a crucial for optimal GC B‐cell responses, coordinating selection and maintenance of high affinity GC B‐cell clones; therefore, we investigated the frequency and activity of the Tfh‐cell subset in the substrains.^40^ The number of Tfh cells induced in response to NP‐OVA in IFA immunization was significantly reduced in low‐responder BALB/c A (Figure 4a), and the expression of the Tfh‐cell lineage‐specific transcription factor Bcl6 and critical B‐cell–stimulatory signals were also reduced (Figure 4b). These data suggest Tfh‐cell activity may also be diminished in low‐responder BALB/c A mice. T follicular regulatory (Tfr) cells have previously been shown to control the Tfh‐cell response.^40^ However, numbers of Tfr cells were significantly reduced in low‐responder BALB/c A mice, indicating Tfr cells were unlikely to be causative of the attenuated Tfh‐cell frequency in this substrain (Figure 4c). To ascertain whether the difference in the Tfh‐cell response was specific to this CD4^+^ T‐cell subset, the differentiation of central and effector memory CD4^+^ T‐cell subsets induced in response to NP‐OVA in IFA immunization was assessed and found to be comparable between the substrains (Figure 4d). Numbers of CD4^+^ T cells in the lymph nodes and spleen were also equivalent under homeostatic conditions (Supplementary figure 3). Furthermore, naïve CD4^+^ T cells were equally able to respond to polyclonal activation *in vitro* (Figure 4e). These results suggest the difference in the Tfh‐cell response is specific to this CD4^+^ T‐cell subset and not due to dysfunction of the CD4^+^ T‐cell compartment as a whole.

### Diminished capability of low‐responder BALB/c A B cells to class switch when stimulated through TLR or CD40 *in vitro*

**Figure 5:**
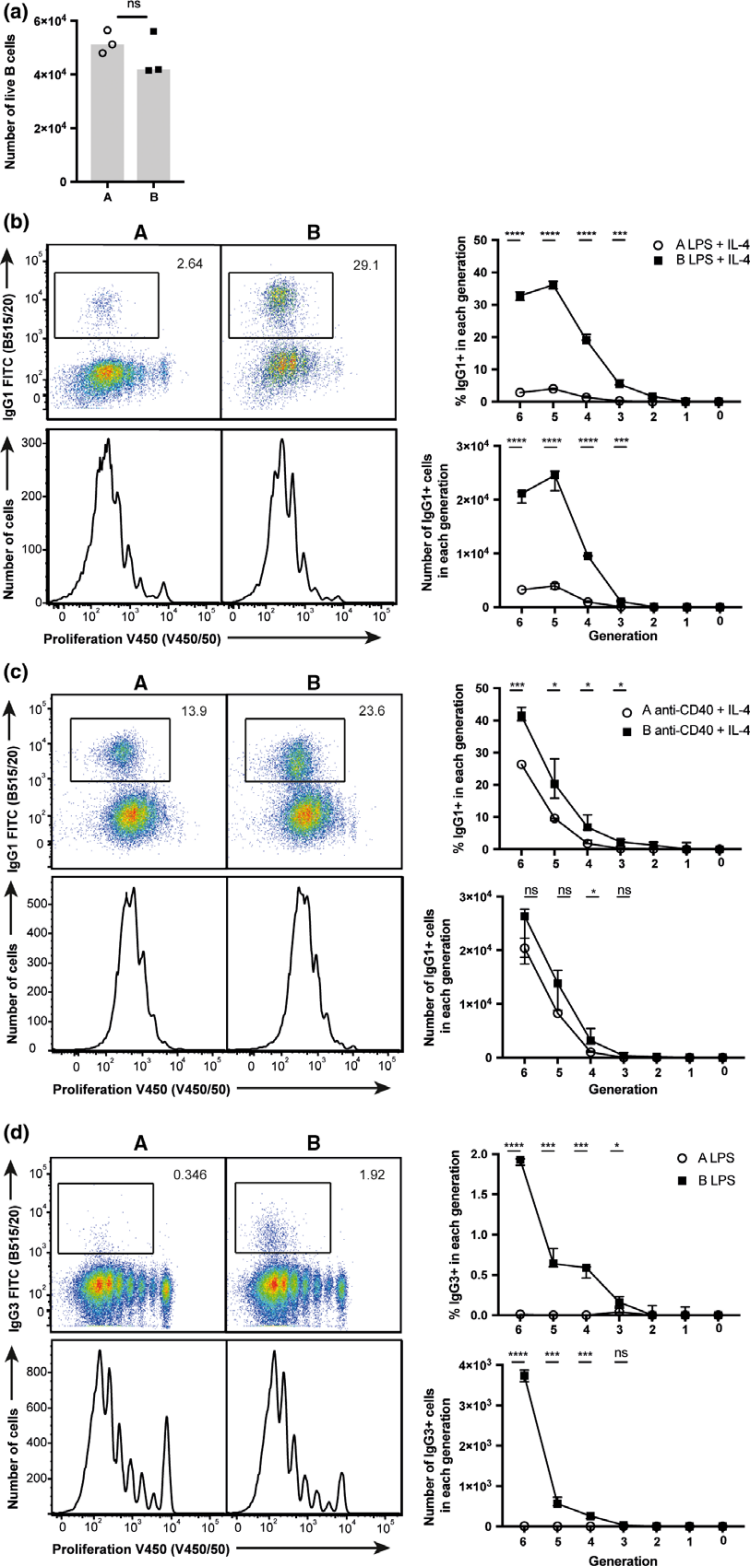
Isotype class switching is defective in BALB/c A B cells. Naïve splenic B cells of BALB/c A and BALB/c B mice were stimulated with LPS + IL‐4 and monitored for **(a)** survival at 24 h and **(b)** C‐S to IgG1 and proliferation at 90 h by flow cytometry; graphs show the frequency and number of IgG1+ cells in each generation gated on size, viability B220+ CD138+ CTV IgM– IgG1+. **(c)** Naïve splenic B cells were stimulated with αCD40 + IL‐4 and monitored for proliferation and C‐S to IgG1 at 90 h by flow cytometry; graphs show the frequency and number of IgG1+ cells in each generation gated on size, viability B220+ CD138+ CTV IgM– IgG1+. **(d)** Naïve splenic B cells were stimulated with LPS and monitored for proliferation and C‐S to IgG3 at 90 h by flow cytometry; graphs show the frequency and number of IgG3+ cells in each generation gated on size, viability B220+ CD138+ CTV IgM– IgG3+. **(a)** Each data point represents one sample. **(b–d)** Representative dot plots from one sample per group and data from triplicate cultures were graphed. Statistical significance was determined using an unpaired *t*‐test. Data are representative of at least three experiments.

We performed quantitative analysis of naïve B cells stimulated *in vitro* to establish whether the altered CSR capability was driven by a B‐cell intrinsic mechanism. Lipopolysaccharide (LPS) + interleukin (IL)‐4 induces B‐cell activation and IgG1 production.^21^, ^41^, ^42^ Viability as measured by total live NIR™ viability stain negative cell numbers at 24 h (Figure 5a) was equivalent between B cells from both mice, demonstrating differences in survival in the context of T‐independent stimulation (LPS + IL‐4) do not account for the difference in antibody responsiveness. Strikingly, IgG1 class switching was significantly reduced in low‐responder BALB/c A B cells despite equivalent proliferation profiles (as measured by dilution of division tracking dye) in B cells of BALB/c A and B (Figure 5b). C‐S is regulated by division number and cannot occur after commitment to differentiated CD138^+^ plasma cells. For this reason, by altering the generation in which C‐S occurs (or conversely that plasma cell differentiation begins), there can be a profound effect on the capability of B cells to C‐S.^43^ Therefore, we compared the kinetics of IgG1 C‐S and plasma cell differentiation in B cells from the BALB/c substrains. Although the frequency and number of IgG1^+^ cells within each generation is significantly reduced in low‐responder BALB/c A B cells (Figure 5b), the distribution of IgG1‐switched B cells across generation number was equivalent. These data demonstrate that C‐S kinetics were comparable (Supplementary figure 4a). The frequencies of PCs in generations three to five were slightly greater in BALB/c A (Supplementary figure 4a). However, no difference in distribution of the total plasma cell population across generations was seen between the substrains, indicating plasma cell differentiation is initiated and progresses equivalently in both substrains (Supplementary figure 4b, c). In summary, C‐S differentiation of plasma cells were comparable, suggesting the capability to C‐S is not influenced by differences in the kinetics of B‐cell responses.

Crosslinking CD40 using soluble αCD40 antibody IC10 acts as an *in vitro* mimic of T‐dependent activation, and in combination with IL‐4 stimulates C‐S to IgG1.^44^ IgG1 C‐S in low‐responder BALB/c A B cells was reduced despite similar proliferation of the B cells in both substrains (Figure 5c). The B cells from low‐responder BALB/c A class switch to a greater frequency in response to αCD40 + IL‐4 stimulation as compared to LPS + IL‐4 stimulation (Figure 5b, c). Previous investigations have shown a greater dependence on IL‐4 signaling to induce IgG1 C‐S following αCD40 stimulation,^45^ and the increased response to this stimulation in low‐responder BALB/c A B cells may suggest a potential dysfunction within the IL‐4 signaling pathway in this substrain. To assess whether differences in the antibody response were dependent purely on the IL‐4 pathway, B cells were stimulated with LPS alone and C‐S to IgG3 isotype was assessed. Again, proliferation was equivalent between B cells from the substrains. However, C‐S to IgG3 was significantly reduced in low‐responder BALB/c A B cells (Figure 5d), demonstrating an intrinsic alteration in CSR in BALB/c A mice that is IL‐4 signaling pathway independent. These results suggest differences in antibody responses are restricted to a deficiency in CSR, as the proliferation and differentiation of B cells from BALB/c A and BALB/c B mice are equivalent to both T‐dependent and ‐independent stimuli. Finally, mice were immunized with NP‐ficoll to assess T‐cell‐independent C‐S capability *in vivo*. The generation of IgG3 antibody was significantly reduced in low‐responder BALB/c A mice (Supplementary figure 4f), further indicating B cell‐intrinsic regulation of the capability to undergo CSR.

### Identification of genetic polymorphisms that associate with aberrant antibody responsiveness

**Figure 6:**
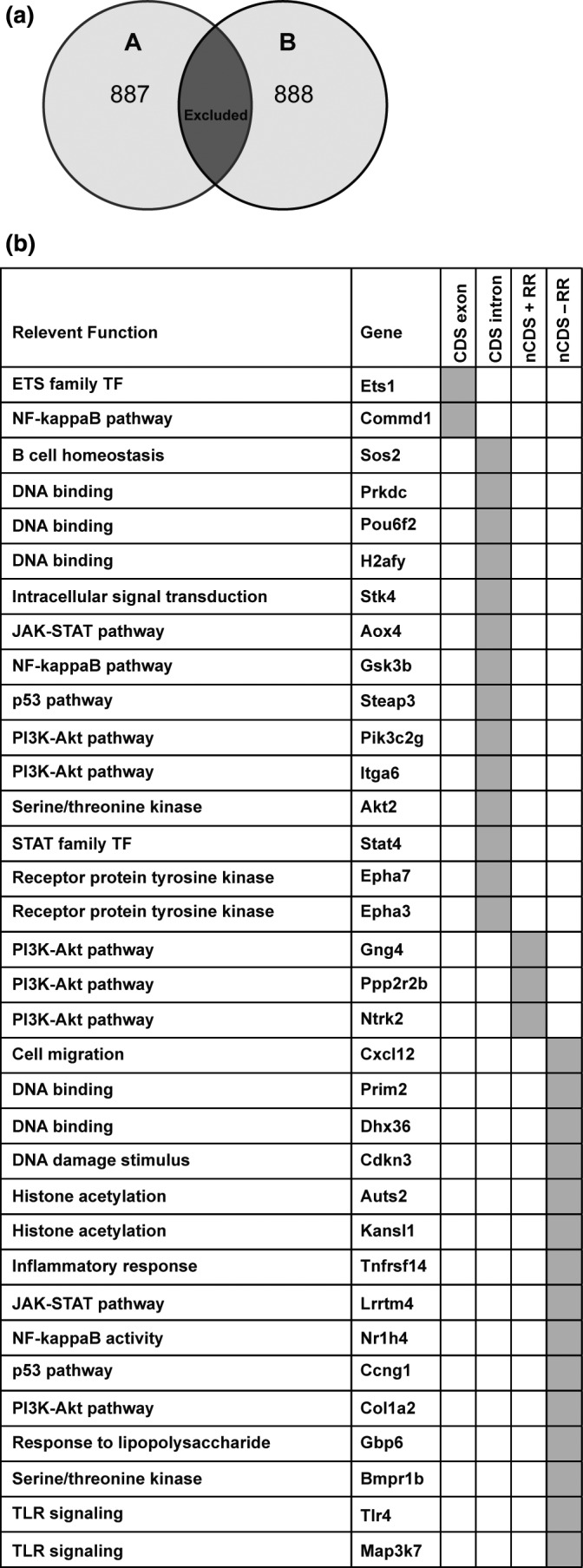
Identification of genetic polymorphisms that associate with altered capability to class‐switch in low‐responder BALB/c A mice. **(a)** The number of SNP and INDEL polymorphisms to the mouse reference genome that are specific to BALB/c A or BALB/c B respectively. **(b)** BALB/c A candidate genes compiled by identification of polymorphisms in known mediators or regulators of CSR. CDS, coding sequence; nCDs, non‐coding sequence; RR, regulatory region.

A whole genome sequencing approach was used to identify genetic polymorphisms that may affect the mediators or regulators of the CSR mechanism in BALB/c A mice. Initially, polymorphisms specific to BALB/c A and BALB/c B were identified (Figure 6a) and a list of variant genes specific to BALB/c A and BALB/c B created through gene assignment to these polymorphisms (Supplementary table 3). We then selected from the list of variant genes specific to BALB/c A, those genes with a known role or ontology linked to the regulation or mechanism of CSR and confirmed their expression in mature B cells using the ImmGen database^23^, ^45^, ^46^, ^47^, ^48^ (Supplementary table 4). A total of 34 gene targets were identified (Figure 6b), and each polymorphism was then interrogated for a predicted effect on protein activity. The Ets1 polymorphism was predicted to influence Ets1‐007 noncoding mRNA, which could potentially affect Ets‐1 protein expression. The Commd1 insertion mutation was predicted to alter regulation of the Commd1‐001 transcript based on its location the 5′ UTR of this transcript. Additionally, we determined that the 14 intronic polymorphisms we had identified had no predicted effect on splicing.

Next, the influence of polymorphism on transcription factor binding sites was predicted using Transfac. The deletion in the regulatory region of protein phosphatase 2, regulatory subunit B, beta (Ppp2r2b) was predicted to result in the loss of three Sox‐4 binding sites, which would likely reduce the transcription of Ppp2r2b as Sox4 is upregulated in activated B cells.^49^ It was predicted that the insertion mutation within the regulatory region of neurotrophic tyrosine kinase, receptor, type 2 (Ntrk2) would result in the addition of a Sox‐4 transcription factor binding site, possibly upregulating its transcription. A deletion in the guanine nucleotide‐binding protein (G protein), gamma 4 (Gng4) regulatory region, was predicted to result in the loss of a Trf1 and RREB1 transcription factor binding site, which may reduce Gng4 transcription. Fifteen further intergenic variants were identified but were not predicted to affect gene activity using our methods. These data propose putative targets in genetic regulation of low antibody responsiveness, and serve as a resource to be mined in the future as more CSR regulators are recognized. Furthermore, the genomic sequences of these two BALB/c A substrains offer a resource for identifying unappreciated regulators of antibody responsiveness through untargeted analytical methods.

## Discussion

In this study, we explored the mechanisms associated with altered antibody responses in substrains of BALB/c mice. We hypothesized this would further our understanding of the factors that influence the heterogeneity of protective antibody titers observed in the human population. Despite the mice harboring dramatically different microbiota compositions, cohousing conferred no alteration on response magnitude. These data indicate that genetic regulation is dominant over environmental influence on antibody responsiveness in these mice. Immunization responsiveness was determined by efficiency of C‐S in GC B cells, which was paralleled by altered Tfh activity. Critical to this study was the identification of a B‐cell intrinsic dysfunction in CSR capability, which was downstream of CD40 and TLR stimulation pathways. This led to the targeted identification of polymorphisms in known regulators or mediators of CSR to elucidate candidate genes that confer the altered immune responsiveness observed between these mice.

It is known that both gene composition and environmental conditions can impact immune responses in mice.^6^, ^32^, ^33^, ^34^, ^35^, ^50^, ^51^ As expected, the gut microbiota composition was substantially different between the substrains of BALB/c mice, but the lack of effect of cohousing on antibody production in either low or high responder BALB/c mice suggests neither the gut microbiota nor other environmental variables were sufficient to drive the antibody responder phenotypes in these mice. Previous findings have demonstrated a lack of microbial regulation in antibody responses induced by adjuvanted vaccines; here, we extend these findings to demonstrate a genetic regulation drives the response capability of these substrains of BALB/c mice.^6^ However, discordant with our results are recent studies demonstrating a dominant role of environment in the regulation of antibody responsiveness in the human population.^8^, ^52^ The discrepancy with our findings may be due to the different ways in which these studies characterized antibody responsiveness. One study assessed influenza‐specific responses induced by diverging frequency and forms of unadjuvented antigen exposure, whereas another correlated total antibodies raised against a multitude of immune challenges. Moreover, in both cases, exposures were over the lifetime of the individual, during which multiple parameters, including age, will impact response magnitude.^8^, ^52^ In turn, our study is a controlled assessment of response capability, measuring antibody magnitude amounted to one adjuvanted antigen exposure. The discrepancy in these data suggest investigations aimed at understanding the relative contribution of environmental and genetic influences on response capability to adjuvanted vaccines and how response capability shapes heterogeneity within populations warrant further investigation. Moreover, it demonstrates microbiota‐targeted approaches to adjuvant immune capability will not be effective in individuals with similar genetic dysfunctions to that observed in low‐responder BALB/c A mice, and suggests immune‐adjuvant approaches may have a broader success rate over a population.

Low‐responder BALB/c A mice have a perturbed B‐cell C‐S capability resulting in a significant reduction in immunization‐induced IgG1, IgG2a and IgE antibody production. Results gained from the *in vitro* stimulation of naïve B cells determined equivalent activation and proliferative capabilities of B cells from both substrains, as well as matched C‐S and plasma cell differentiation kinetics. However, the capability to undergo CSR to IgG1 and IgG3 was affected in low‐responder BALB/c A mice, suggesting a genetic dysfunction afflicts a B‐cell intrinsic regulator or mediator of CSR. Low‐responder BALB/c A mice were able to mount a sufficient GC B‐cell response and appropriate IgM production. However, subsequent isotype class‐switch responses were skewed, leading to significantly reduced IgG1, IgG2a and IgE responses in BALB/c A. As there are no known markers for selection of GC B cells in this transition state, we are unable to characterize the isotype expression profile of this responding population. The Tfh response was also reduced in low‐responder BALB/c A mice indicating that the pathways in the generation of the antibody response, in addition to CSR, are also deficient in BALB/c A. Further investigation is required to determine whether perturbation in Tfh responsiveness and CSR capability are regulated by shared or distinct polymorphisms, and indeed if other immunological mechanisms are also affected. Toward the development of immune‐adjuvant therapeutics, determining the relative contribution that CSR efficiency and Tfh responsiveness impart to the magnitude of antibody response capability could provide valuable insight to whether therapeutic targeting of just one dominant pathway would be sufficient to boost responsiveness, or whether multiple pathways in antibody generation need to be targeted in unison.

The observation that at least some B cells from low‐responder BALB/c A mice retained the capability to class switch suggests the genetic dysfunction afflicts protein activity of a C‐S regulator. Moreover, altered C‐S capability after stimulation through both TLR and CD40 demonstrates the C‐S regulator lies downstream of multiple stimulation pathways and therefore may be a mediator of the CSR mechanism after these pathways converge, or alternatively, it is shared across multiple pathways. Intriguingly, we noted that B cells from low‐responder BALB/c A mice had a greater capacity to undergo C‐S in response to CD40 stimulation compared to TLR ligands. This suggests that the functional class‐switch B‐cell polymorphisms we observed could be more pervasive following T‐cell–dependent stimuli, and germinal center responses. Thus, deciphering the origin of this difference could identify promising adjuvant candidates for improving antibody responses *in vivo*.

Identification of dysfunctional genetic elements in the C‐S mechanism utilizing differential gene expression analysis was deemed unfeasible in the absence of a method to positively isolate cells actively undergoing class switching. Instead, investigation of genetic polymorphisms affecting known regulators or mediators of CSR from whole genome sequence data was performed to elucidate genetic control of the altered C‐S capability of these substrains. Polymorphisms in BALB/c A mice were interrogated as it was deemed likely to be the deviant substrain given the perturbed C‐S capability is at odds to the expected antibody response capability of BALB/c mice.^53^ Four candidates with the greatest potential to confer the perturbed C‐S response capability of low‐responder BALB/c A mice were identified. Altered Ets1 activity has the potential to regulate C‐S to all antibody isotypes, as Ets1 binds to the regulatory region 3′ to Ighα controlling germline transcription of all switch regions.^47^ Ets1 has previously been shown to be critical only for C‐S to IgG2a and not IgG1, IgG2b or IgG3 isotype switching using an Ets‐1^−/−^ chimera approach, which is at odds with reduced C‐S to all isotypes observed in low‐responder BALB/c A.^54^ However, altered protein activity may induce a different effect on C‐S as compared to protein absence as was modeled with the Ets‐1^−/−^ mice, and therefore, Ets1 remains a strong candidate for conferring the BALB/c A class‐switch phenotype. The other three key candidate polymorphisms are all located in regulatory regions of phosphatylinositol 3‐kinase (PI3K) signaling mediators. PI3K‐Akt signaling controls activation‐induced cytidine deaminase expression and GC B‐cell phenotype through tight regulation of forkhead box O proteins (FOXO), with enhanced PI3K‐Akt signaling inhibiting FOXO activation of activation‐induced cytidine deaminase‐driven C‐S.^55^ The polymorphisms affecting Ppp2r2b and Ntrk2 act to enhance PI3K‐Akt signaling and thus potentially suppress FOXO‐driven activation of activation‐induced cytidine deaminase and C‐S in BALB/c A. The final candidate Gng4 is a mediator of CXCR4 signaling to the PI3k‐Akt pathway;^56^ however, recent demonstration that CSR capability is intact in CXCR4^−/−^ GC B cells would challenge the potential impact of this polymorphism.^57^ Notably, the lack of clear support for any of these four candidates as likely mediators of the low‐responder phenotype in BALB/c A mice indicates that a unique or previously unappreciated regulator of the CSR mechanism may perturb C‐S capability in BALB/c A B cells. It is also possible that epigenetic regulation may play a role; therefore, the validation that known mediators or regulators of CSR are not perturbed in this way is imperative.^58^ In addition to the perturbed response in BALB/c A mice, it remains to be determined whether SNPs in BALB/c B mice may alternatively confer a heightened antibody response. These results demonstrate that although we have sound understanding of the T‐ and B‐cell mechanisms that regulate antibody responsiveness, there are still gaps in our knowledge regarding genetic mechanisms controlling C‐S capability.

In summary, this study has identified a B‐cell intrinsic genetic dysfunction that perturbs antibody response capability. These investigations are timely, given the recent data supporting a stronger role for environmental influences on B‐cell responses and antibody production.^4^, ^8^, ^52^, ^59^ These discrepancies may well be resolved when immune‐adjuvant or microbiota‐targeted approaches to boost antibody responsiveness in the human population are compared across both unadjuvanted and adjuvanted vaccine responses.^6^ Based on a lack of clear genetic polymorphisms in known CSR candidate genes in low‐responder BALB/c A mice, we attribute this altered antibody response capability to a potentially novel or previously unappreciated regulator of CSR. This study also provides a genome sequence resource that could be exploited to identify this unknown regulator, and potentially elucidate polymorphisms shared with low responders in the human population. If successful, low‐responder BALB/c A mice will be a useful tool to explore new immune adjuvants to augment antibody responsiveness and boost vaccine efficacy.

## Methods

### Mice, cohousing and bone marrow chimeras

Specific pathogen‐free BALB/c (BALB/c A) were originally imported to the Malaghan Institute from the Animal Resources Centre, Murdoch, Australia in January 2010 and BALB/c By (BALB/c B) were originally imported to the Malaghan Institute from the Jackson Laboratories, Bar Harbour, Maine, USA, in May 2011. Mice were bred and housed at the Malaghan Institute of Medical Research Biomedical Research Unit and were utilized for this study between December 2013 and March 2017, with BALB/c A in generation N18 and BALB/c B generation N11 at time of completion of all experiments. Age‐ and sex‐matched mice were used at 6–8 weeks of age unless otherwise indicated. To promote microbiota transfer in specified experiments, 4‐week‐old mice were cohoused for 4 weeks prior to immunization. Fecal samples were collected after 4 weeks of cohousing and mice were vaccinated the day after fecal sample collection. Cohousing was maintained for a further 2 weeks until the endpoint of the experiment.

### Infections and immunizations

Immunizations were administered subcutaneously. IFA (Sigma‐Aldrich, Auckland, New Zealand) was formulated at 1:1 volume with 50 μg of ovalbumin (OVA; Sigma‐Aldrich) or NP‐OVA [4‐hydroxy‐3‐nitrophenyl (acetyl coupled to OVA); Biosearch Technologies, Novato, CA, USA] in phosphate buffered saline. In place of IFA, 1 mg of Alum was used in specified experiments (Alu‐gel‐S, Serva Electrophoresis GmbH, Heidelberg, Germany). Unadjuvanted trivalent seasonal influenza vaccine (Influvac, AbbotBiologicals, Macquarie Park, Australia) was used at 1:10 of the human dose.

### Enzyme‐linked immunosorbent assay

Commercial kits were used to quantify total serum IgG1 (eBioscience, San Diego, CA, USA), IgG2a (eBioscience), IgE (BD Biosciences, San Jose, CA, USA) and antinuclear antigen antibodies; IgG, IgA and IgM reactive to DNA, SSA/Ro, SSB/La, Scl70, Sm, RNP, Jo‐1 (Alpha Diagnostic International, San Antonio, TX, USA). Antibodies used in noncommercial ELISAs are listed in Supplementary table 1. Serum NP‐specific antibody levels were determined with NP coupled to bovine serum albumin (NP‐BSA; Biosearch Technologies) for capture followed by biotinylated anti‐mouse IgG1, IgG2a, IgE or IgM detection. Plates were then incubated with streptavidin‐HRP (Biolegend, San Diego, CA, USA), followed by TMB substrate (BD Biosciences) for colorimetric development. Plates were analyzed at 450 nm and graphs display the total OD450 reading with a dotted line representing background.

### Cell isolation

Single cell suspensions were prepared from the lymph nodes, spleens and bone marrow by mechanical disruption and passage through a 70‐μm nylon strainer (BD Biosciences). Red blood cell lysis of splenocytes was performed with tris‐buffered ammonium chloride for 5 min at room temperature.

### *In vitro* cell culture

Purified B cells were isolated following a previously described method.^44^ Briefly, purified B cells were isolated for *in vitro* stimulation from RBC‐lysed splenocytes first separated by a density step gradient of 50, 65 and 80% Percoll PLUS (Sigma‐Aldrich) diluted with phosphate buffered saline. Cells at the 65 and 80% interface were collected and B cells isolated using the MACS B cell isolation kit (Miltenyi Biotec, Bergisch Gladbach, Germany). Purified B cells were cultured at 2 × 10^5^ cells mL^−1^ in Iscove's modified Dulbecco medium + GlutaMAX (Sigma‐Aldrich) supplemented with fetal bovine serum (Sigma‐Aldrich), penicillin/streptomycin (Sigma‐Aldrich) and β‐mercaptoethanol (Sigma‐Aldrich) with or without stimulants; LPS (L2654; Sigma‐Aldrich) 15 μg mL^−1^, IL‐4 (generated from CHO‐IL4 hybridoma cells) 1000 U mL^−1^ and recombinant IL‐5 (Biolegend) 1000 U mL^−1^. CD4 T cells were isolated from mesenteric lymph nodes using the MACS CD4 T cell isolation kit (Miltenyi Biotec) and cultured in Iscove's modified Dulbecco medium plus GlutaMAX (Sigma‐Aldrich) supplemented with fetal bovine serum (Sigma‐Aldrich) and penicillin/streptomycin (Sigma‐Aldrich), with the addition of anti‐CD28 (generated from 37.51 hybridoma cells) with or without IL‐2, 100 U mL^−1^ (Proleukin, Chiron, Emeryville, CA, USA). Cells were plated at 2 × 10^5^ cells mL^−1^ in 96‐well plates coated with 10 μg mL^−1^ anti‐CD3 (generated from 2C11 hybridoma cells).

### Antibodies and flow cytometry

Cells were stained with zombie NIR™ fixable viability stain (Biolegend) at 4°C for 10 min to identify dead cells. Cell surface antibodies and anti‐mouse CD16/32 (generated from 24G2 hybridoma cells) were diluted in phosphate buffered saline with 2% fetal bovine serum and incubated at room temperature for 30 min. The FoxP3 fixation and permeabilization buffer set (eBioscience) was used when staining for intracellular markers. Antibodies and anti‐mouse CD16/32 were diluted in permeabilization buffer and incubated at room temperature for 30 min. To assess proliferation, 5 x 10^5 ^cells mL^−1^ were stained with CellTrace Violet (Life Technologies) for 4 min at 37°C. Staining was stopped by the addition of fetal bovine serum and cells washed four times in Iscove's modified Dulbecco medium supplemented with fetal bovine serum (Sigma‐Aldrich). Samples were acquired on an LSRII or Fortessa cytometer (BD Biosciences) linked to FACSDIVA software (BD Biosciences) and analyzed with Flow Jo v9 (Tree Star Inc, CA, USA). In sorting experiments, cells were isolated using a FACSVantage (BD Biosciences) linked to FACSDIVA software (BD Biosciences). Antibodies used for flow cytometry including cell sorting are listed in Supplementary table 1.

### Immunohistochemistry

Antibodies used for immunohistochemistry are listed in Supplementary table 1. Lymph nodes were snap frozen in OCT Tissue‐Tek OCT compound (Sakura Finetek, the Netherlands). Six 5‐μm frozen sections per sample were taken from the efferent lymph vessel at an interval of 50 μm. Follicular B cells were detected with B220 and germinal center B cells detected with GL7. The T‐cell zone was visualized with CD4. Stained sections were mounted with KPL fluorescent mounting media (Gaithersberg, MD, USA) and analyzed with a laser‐scanning Olympus FV1200 confocal microscope (Tokyo, Japan) using a 10× objective.

### Quantitative real‐time PCR

RNA was isolated from sorted cells using the Arcturus Picopure RNA Isolation Kit (Applied Biosystems, Foster City, CA, USA) following the manufacturer instructions. Briefly, cells were sheared with a needle then lysed in extraction buffer at 42°C, the supernatant containing extracted RNA was then loaded onto RNA purification columns, washed and eluted in 20 μL of RNAse‐free water. RNA was converted to cDNA using the High‐capacity cDNA Reverse Transcription Kit (Applied Biosystems) following the manufacturer's instructions. Quantitative real‐time PCR was performed with specific Taqman primers (Life Technologies) and the Taqman fast Universal PCR master mix (Applied Biosystems) on the QuantStudio 7 flex platform (Life Techniologies). Primers used for qRT‐PCR are listed in Supplementary table 2.

### 16s ribosomal RNA Illumina sequencing and analysis

For microbiota profiling, genomic DNA was extracted from fecal pellets using the DNA mini stool kit (Qiagen, Hilden, Germany) following the manufacturer's instructions. Briefly, each fecal sample was lysed with proteinase K and lysis buffer at 70°C, and the lysed suspension was then loaded onto a QIAmp spin columns, washed and eluted in 100 μL of RNAse‐free water. DNA yield was assessed using the Quantus™ fluorometer (Promega, Madison, WA, USA), and DNA quality was measured with the Nanodrop ND‐1000 spectrophotometer (Thermofisher, Waltham, MA, USA). Amplification of the V3–V4 regions of the 16S rRNA gene followed by 2 × 250 bp sequencing on the MiSeq platform was performed at NZ Genomics Ltd (NZGL) using the standard Illumina method (Amplicon PCR primers IP of Illumina) https://support.illumina.com/content/dam/illumina-support/documents/documentation/chemistry_documentation/16s/16s-metagenomic-library-prep-guide-15044223-b.pdf. Amplicon sequences were processed using Qiime 1.8. Paired end reads were quality filtered using a Q30 cutoff, and chimeric sequences identified using the USEARCH method against the Greengenes alignment (version 13_8) were removed. OTUs were picked at 97% similarity using the UCLUST method, and representative sequences were assigned taxonomies using the RDP classifier. Principle component analysis of the relative abundances of taxa identified up to the genus level for Balb/c, Balb/c A^ch^, Balb/c B and Balb/c B^ch^.

### Whole genome sequencing and analysis

For whole genome sequencing, genomic DNA was extracted from 100 mg of fresh liver tissue using the DNeasy Tissue kit (Qiagen) following the manufacturer instructions. Briefly, each liver tissue sample was lysed with proteinase K and lysis buffer and incubated over night at 55°C, the lysed tissue was then loaded onto a DNeasy spin columns with a silica‐gel membrane, washed and eluted in 100 μL elution buffer. DNA yield was assessed using the Quantus™ fluorometer (Promega) and DNA quality was measured using the Nanodrop ND‐1000 spectrophotometer (Thermofisher). A quantity of 1 μg of liver DNA was sheared and then PE sequencing libraries were constructed using an Illumina TruSeq DNA Sample Preparation kit (Thermofisher) according to the manufacturer's recommended protocol. The library was quantified using Qubit fluorometry (Thermofisher) and sized on an Agilent Bioanalyser DNA chip (Agilent Technologies, Santa Clara, CA, USA). The resulting whole genome library was amplified on a flow cell using an Illumina cBot cluster station (Illumina, San Diego, CA, USA) and 350 million paired end reads at 125 bp length were generated on an Illumina HiSeq 1000 at NZGL. The sequences have been uploaded to the SRA database and are awaiting approval, submission number SUB2635230.

Quality filtering was performed using trimmomatic with a sliding window of 10 nt, quality ≥15 and read length ≥120 nt (Bolger Bioinf, 2014). The remaining high quality reads were mapped at 99% similarity to the mouse reference genome (GRCm38.p5) using BBMap with default parameters (https://sourceforge.net/projects/bbmap/). SNP and INDEL polymorphisms to the reference genome (GRCm38.p5) were called using samtools and bcftools.^60^ Variants were filtered using a custom Ruby script keeping only those with a score >100, >10 reads mapped to the variant allele, >10 nt to each other and not present in the other line (A *vs* B) to give list of polymorphisms specific to BALB/c A and a list specific to BALB/c B. Mutations existed throughout the genome and were assigned to neighboring genes if not in coding sequence using a custom Perl script that matched the genomic position of the variant to genes described in the genome feature table GCF_000001635.25_GRCm38.p5 from ensemble.

Bioinformatic analysis was performed to identify a list of previously reported relevant genes in signaling pathways (KEGG pathway database mmu04662, mmu04620, mmu04630, mmu04151, mmu04064, mmu04115). Gene ontology assignment to variant genes was performed with the MGI database. Immgen was used to assess expression patterns (Gene skyline database, key populations and B cell data group). The predicted effect of polymorphisms was determined using various databases: genome location: MGI mouse genome browser (Jbrowse), VEGA gene model and NCBI databases; splicing prediction: Human splice finder, AAS sites and Sroogle^61^, ^62^; regulatory region location: ORegAnno within UCSC Genome Browser Mouse Dec. 2011 (GRCm38/mm10) genome assembly; and transcription factor binding: Transfac databases (vertebrate_non_redundant_minFP and immune_specific).

### Statistical analysis

Single comparisons were made using the Mann–Whitney *U*‐test unless otherwise stated. All statistical analyses were performed using GraphPad Prism v6: *P ≤ 0.05, **P ≤ 0.01, ***P ≤ 0.001 and ****P ≤ 0.0001.
