# Supplementary material for: Genetic regulation of antibody responsiveness to immunization in substrains of BALB/c mice
Source: Immunol Cell Biol. 2018 Oct 14;97(1):39–53. doi: 10.1111/imcb.12199 (PMC6378622; doi:10.1111/imcb.12199)
Supplement: Supplementary file 1 [file IMCB-97-39-s001.docx]

**Supplementary Figure 1**

**
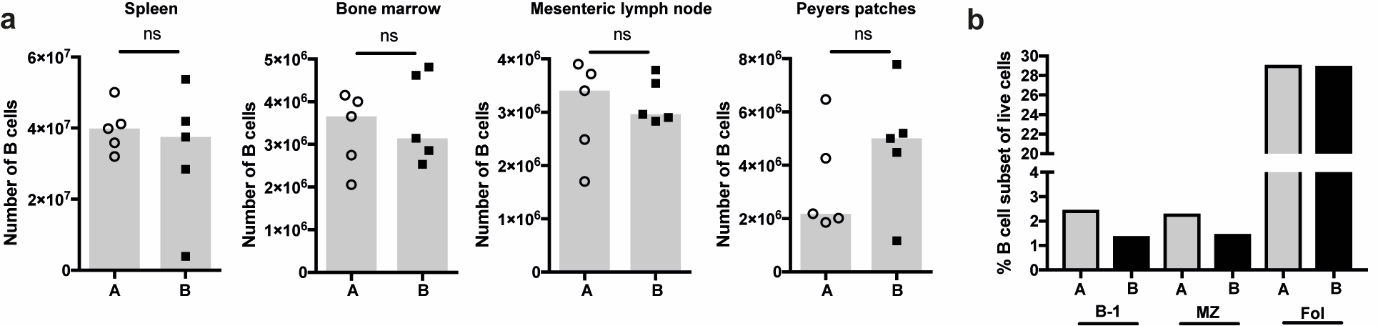
**

**Frequencies of B cell subsets in naïve 6-8 week old BALB/c A and B mice assessed by flow cytometry.**

(**a**) Number of B cells in specified tissues gated as live B220+ CD138-. Data points represent individual mice and heights of the bar the median. Statistical significance determined using the Mann-Whitney *U*-test. (**b**) Frequencies of B-1, marginal zone (MZ) and follicular (Fol) B cell subsets within the spleen as a percentage of live cells. Each bar represents data from three pooled spleens. Gated as: B-1 = live CD43+ CD19+, MZ = live CD43-CD19+CD1d+, Fol = live CD43-CD19+CD1d-
