# Supplementary material for: Genetic regulation of antibody responsiveness to immunization in substrains of BALB/c mice
Source: Immunol Cell Biol. 2018 Oct 14;97(1):39–53. doi: 10.1111/imcb.12199 (PMC6378622; doi:10.1111/imcb.12199)
Supplement: Supplementary file 2 [file IMCB-97-39-s002.docx]

**Supplementary Figure 2**


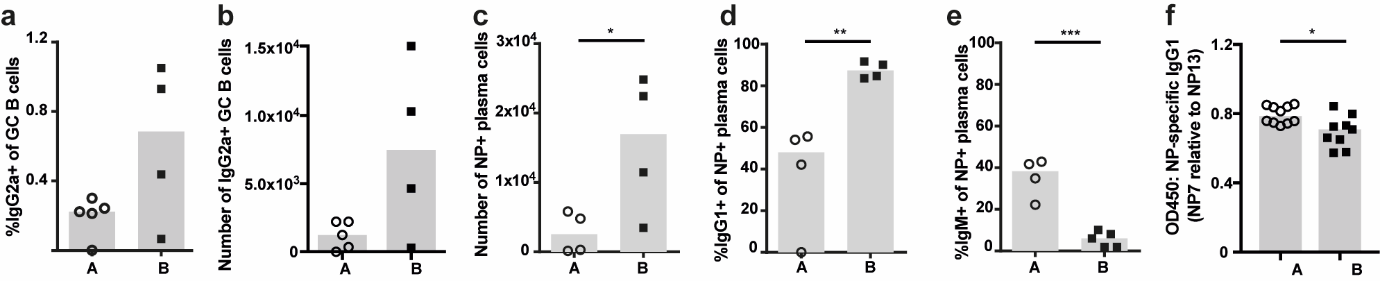


**Efficiency of IgG2a+ GC B cell generation and somatic hypermutation in response to NP-OVA+IFA immunization.**

(**a**) The frequency and (**b**) number of IgG2a+ GC B cells, (**c**) number of NP+ plasma cells, (**d**) frequency of IgG1+ and (**e**) IgM+ NP+ plasma cells in draining lymph nodes of BALB/c A and B mice 14 days after subcutaneous NP-OVA+IFA vaccination was determined using flow cytometry. (**f**) Binding capability of NP-specific IgG1 antibodies to NP at a conjugation ratio of 7 and 13 was determined by ELISA. The ratio of OD450 values from NP7 over NP13 is displayed as a readout of antibody affinity generated by BALB/c A and B. Data points represent individual mice and heights of the bar the median.
