# Supplementary material for: Genetic regulation of antibody responsiveness to immunization in substrains of BALB/c mice
Source: Immunol Cell Biol. 2018 Oct 14;97(1):39–53. doi: 10.1111/imcb.12199 (PMC6378622; doi:10.1111/imcb.12199)
Supplement: Supplementary file 3 [file IMCB-97-39-s003.docx]

**Supplementary Figure 3**


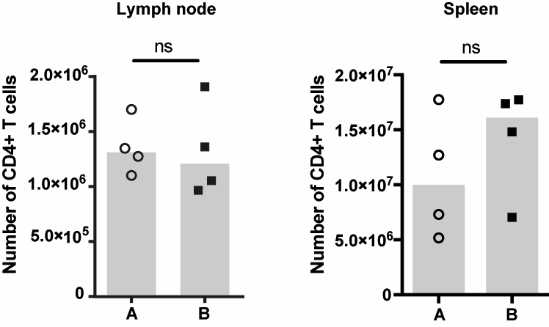


**Baseline frequencies of CD4+ T cells in naïve BALB/c A and B mice.**

CD4+ T cell frequency in naïve 6-8 week old BALB/c A and B mice assessed by flow cytometry. Number CD4+ T cells in (**a**) lymph node and (**b**) spleen. Data points represent individual mice and heights of the bar the median. Statistical significance determined using the Mann-Whitney *U*-test.
