# Supplementary material for: Genetic regulation of antibody responsiveness to immunization in substrains of BALB/c mice
Source: Immunol Cell Biol. 2018 Oct 14;97(1):39–53. doi: 10.1111/imcb.12199 (PMC6378622; doi:10.1111/imcb.12199)
Supplement: Supplementary file 4 [file IMCB-97-39-s004.docx]

**Supplementary Figure 4**


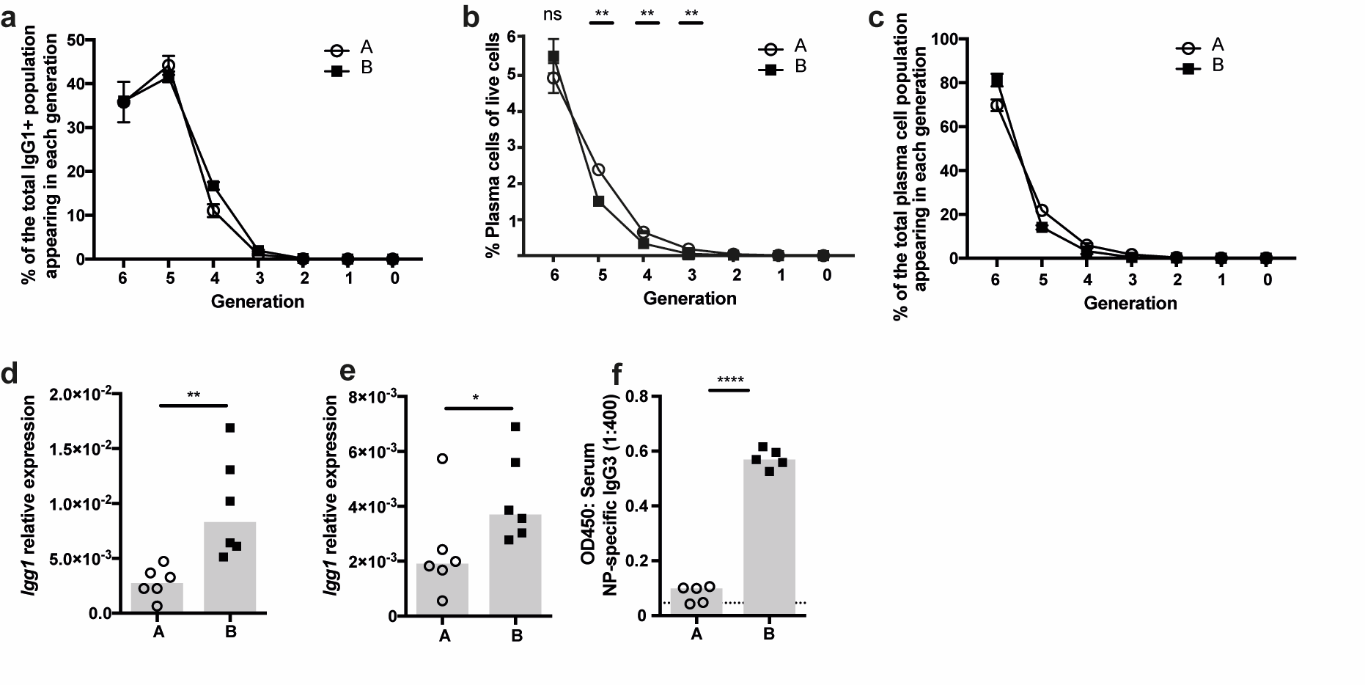


**Determining the kinetic of IgG1 class-switch, plasma cell differentiation and IgG1 transcription in B cells stimulated *in vitro*.**

Naïve B cells stimulated in vitro were assessed at 90 hours for proliferation and C-S to IgG1 by flow cytometry or at 168 hours for IgG1 and 18s mRNA quantity by qRT-PCR. (**a**) Stimulation with LPS + IL-4; percent of IgG1+ cells in each generation. (**b**) Stimulation with LPS + IL-4 + IL-5; CD138+ cells as a percent of live cells across each generation and (**c**) percent of total CD138+ population in each generation. Data from triplicate samples is graphed. (**d**) Stimulation with LPS + IL-4 + IL-5; expression of IgG1 relative to 18s. (**e**) Stimulation with anti-CD40 + IL-4; expression of IgG1 relative to 18s. Data points represent one culture well and heights of the bar the median. (**f**) NP-specific IgG3 serum antibody titers 28 days after immunization with NP-ficoll measured by ELISA. Data points represent individual mice and heights of the bar represent the median. Dashed lines represent lower limit of sensitivity, set at blank OD. Statistical significance determined using the Mann-Whitney *U*-test.
