# Supplementary material for: Genetic regulation of antibody responsiveness to immunization in substrains of BALB/c mice
Source: Immunol Cell Biol. 2018 Oct 14;97(1):39–53. doi: 10.1111/imcb.12199 (PMC6378622; doi:10.1111/imcb.12199)
Supplement: Supplementary file 5 [file IMCB-97-39-s005.docx]

**Supplementary table 1**

Antibodies used for ELISA, flow cytometry and immunohistochemistry

| **Target** | **Clone** | **Conjugate** | **Supplier** |
| --- | --- | --- | --- |
| **ELISA** |  |  |  |
| Anti-mouse IgG1 | LO-MG1-2 | Biotinylated | Life Technologies, Carlsbad, CA, USA |
| Anti-mouse IgG2a | LO-MG2a-3 | Biotinylated | Life Technologies |
| Anti-mouse IgE | R35-118 | Biotinylated | BD Biosciences, San Jose, CA, USA |
| Anti-mouse IgM | II/41 | Biotinylated | BD |
| **Flow cytometry** |  |  |  |
| Anti-mouse CXCR5 | 2G8 | APC | BD |
| Anti-mouse CD4 | RM4-5 | BV605 | BD |
| Anti-mouse CD279 | RMPI-30 | Percpef710 | eBioscience, San Diego, CA, USA |
| Anti-mouse CD90.1 | OX-7 | BUV737 | BD |
| Anti-mouse CD90.2 | 53-2.1 | BUV395 | BD |
| Anti-mouse CD3 | 145-2C11 | BV786 | BD |
| Anti-mouse CD44 | IM7 | AF700 | Biolegend, San Diego CA, USA |
| Anti-mouse FoxP3 | FJK-16s | PE | eBioscience |
| Anti-mouse CD138 | 281-2 | BUV737 | BD |
| Anti-mouse B220 | DX5 | PECF594 | BD |
| Anti-mouse CD38 | 90 | PECy7 | Biolegend |
| Anti-mouse GL7 | GL7 | FITC | Biolegend |
| Anti-mouse GL7 | GL7 | BV421 | Biolegend |
| Anti-mouse IgD | 11-26c.2a | BV710 | Biolegend |
| Anti-mouse IgM | II/41 | APC | BD |
| Anti-mouse IgG1 | RMG1-1 | BV421 | Biolegend |
| Anti-mouse IgG2a | RMG2a-62 | FITC | Biolegend |
| NP-PE |  | PE | Biosearch Technologies, Novato, CA, USA |
| **Immunohistochemistry** |  |  |  |
| Anti-mouse B220 | RA3-6B2 | AF594 | Biolegend |
| Anti-mouse GL7 | GL7 | BV421 | Biolegend |
| Anti-mouse CD4 | RM4-5 | APC | BD |
|  |  |  |  |
