# Supplementary material for: Genetic regulation of antibody responsiveness to immunization in substrains of BALB/c mice
Source: Immunol Cell Biol. 2018 Oct 14;97(1):39–53. doi: 10.1111/imcb.12199 (PMC6378622; doi:10.1111/imcb.12199)
Supplement: Supplementary file 6 [file IMCB-97-39-s006.docx]

**Supplementary table 2**

Primers used for qRT-PCR

| **Target** | **Product code** | **Supplier** |
| --- | --- | --- |
| Igg1 | Mm01742100_s1 | Life Technologies, Carlsbad, CA, USA |
| Bcl6 | Mm00477633_m1 | Life Technologies |
| Il4 | Mm00445259_m1 | Life Technologies |
| Il21 | Mm00517640_m1 | Life Technologies |
| Cd40l | Mm00441911_m1 | Life Technologies |
|  |  |  |
