# Supplementary material for: Genetic regulation of antibody responsiveness to immunization in substrains of BALB/c mice
Source: Immunol Cell Biol. 2018 Oct 14;97(1):39–53. doi: 10.1111/imcb.12199 (PMC6378622; doi:10.1111/imcb.12199)
Supplement: Supplementary file 9 [file IMCB-97-39-s009.pdf]

Supplementary Figure 1

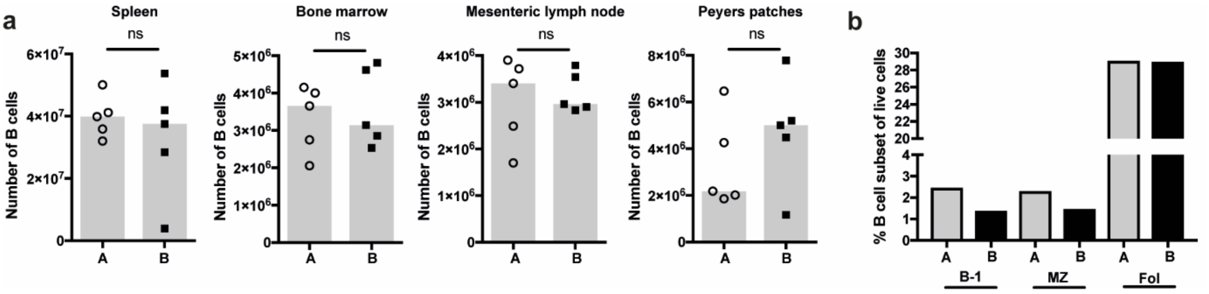

Frequencies of B cell subsets in naïve 6-8 week old BALB/c A and B mice assessed by flow cytometry.

(**a**) Number of B cells in specified tissues gated as live B220+ CD138-. Data points represent individual mice and heights of the bar the median. Statistical significance determined using the Mann-Whitney *U*-test. (**b**) Frequencies of B-1, marginal zone (MZ) and follicular (Fol) B cell subsets within the spleen as a percentage of live cells. Each bar represents data from three pooled spleens. Gated as: B-1 = live CD43+ CD19+, MZ = live CD43-CD19+CD1d+, Fol = live CD43-CD19+CD1d-

Supplementary Figure 2

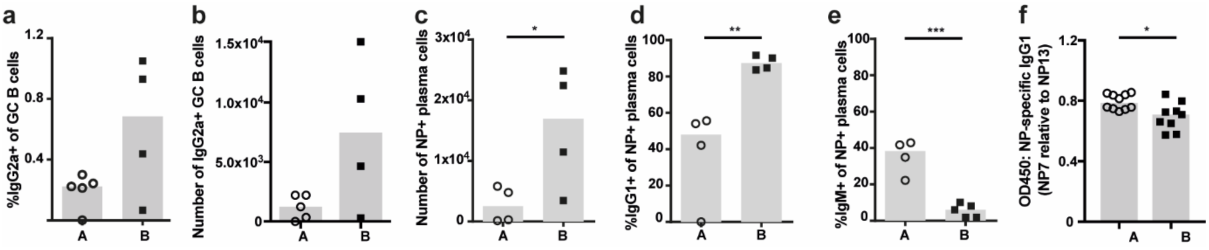

Efficiency of IgG2a+ GC B cell generation and somatic hypermutation in response to NP-OVA+IFA immunization.

(**a**) The frequency and (**b**) number of IgG2a+ GC B cells, (**c**) number of NP+ plasma cells, (**d**) frequency of IgG1+ and (**e**) IgM+ NP+ plasma cells in draining lymph nodes of BALB/c A and B mice 14 days after subcutaneous NP-OVA+IFA vaccination was determined using flow cytometry. (**f**) Binding capability of NP-specific IgG1 antibodies to NP at a conjugation ratio of 7 and 13 was determined by ELISA. The ratio of OD450 values from NP7 over NP13 is displayed as a readout of antibody affinity generated by BALB/c A and B. Data points represent individual mice and heights of the bar the median.

Supplementary Figure 3

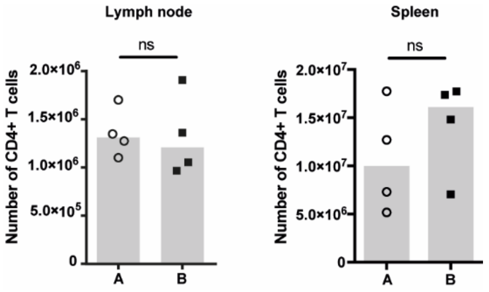

Baseline frequencies of CD4+ T cells in naïve BALB/c A and B mice.

CD4+ T cell frequency in naïve 6-8 week old BALB/c A and B mice assessed by flow cytometry. Number CD4+ T cells in (**a**) lymph node and (**b**) spleen. Data points represent individual mice and heights of the bar the median. Statistical significance determined using the Mann-Whitney *U*-test.

Supplementary Figure 4

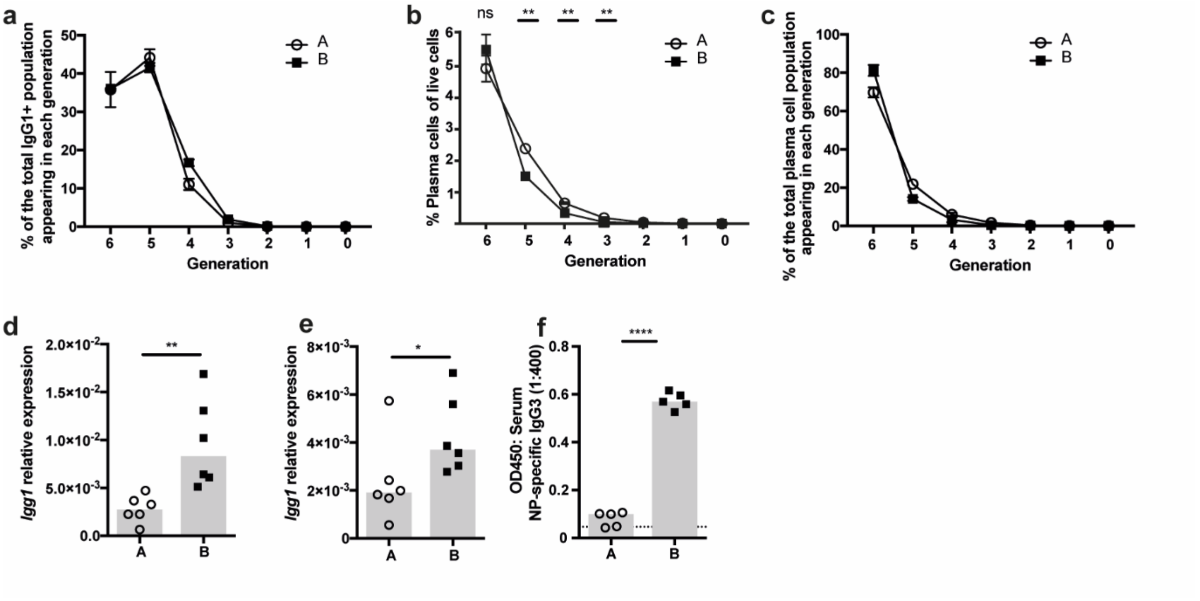

Determining the kinetic of IgG1 class-switch, plasma cell differentiation and IgG1 transcription in B cells stimulated *in vitro*.

Naïve B cells stimulated in vitro were assessed at 90 hours for proliferation and C-S to IgG1 by flow cytometry or at 168 hours for IgG1 and 18s mRNA quantity by qRT-PCR. (**a**) Stimulation with LPS + IL-4; percent of IgG1+ cells in each generation. (**b**) Stimulation with LPS + IL-4 + IL-5; CD138+ cells as a percent of live cells across each generation and (**c**) percent of total CD138+ population in each generation. Data from triplicate samples is graphed. (**d**) Stimulation with LPS + IL-4 + IL-5; expression of IgG1 relative to 18s. (**e**) Stimulation with anti-CD40 + IL-4; expression of IgG1 relative to 18s. Data points represent one culture well and heights of the bar the median. (**f**) NP-specific IgG3 serum antibody titers 28 days after immunization with NP-ficoll measured by ELISA. Data points represent individual mice and heights of the bar represent the median. Dashed lines represent lower limit of sensitivity, set at blank OD. Statistical significance determined using the Mann-Whitney *U*-test.

Supplementary table 1

Antibodies used for ELISA, flow cytometry and immunohistochemistry

Target	Clone	Conjugate	Supplier
**ELISA**			
Anti-mouse IgG1	LO-MG1-2	Biotinylated	Life Technologies, Carlsbad, CA, USA
Anti-mouse IgG2a	LO-MG2a-3	Biotinylated	Life Technologies
Anti-mouse IgE	R35-118	Biotinylated	BD Biosciences, San Jose, CA, USA
Anti-mouse IgM	II/41	Biotinylated	BD
**Flow cytometry**			
Anti-mouse CXCR5	2G8	APC	BD
Anti-mouse CD4	RM4-5	BV605	BD
Anti-mouse CD279	RMPI-30	Percpef710	eBioscience, San Diego, CA, USA
Anti-mouse CD90.1	OX-7	BUV737	BD
Anti-mouse CD90.2	53-2.1	BUV395	BD
Anti-mouse CD3	145-2C11	BV786	BD
Anti-mouse CD44	IM7	AF700	Biolegend, San Diego CA, USA
Anti-mouse FoxP3	FJK-16s	PE	eBioscience
Anti-mouse CD138	281-2	BUV737	BD
Anti-mouse B220	DX5	PECF594	BD
Anti-mouse CD38	90	PECy7	Biolegend
Anti-mouse GL7	GL7	FITC	Biolegend
Anti-mouse GL7	GL7	BV421	Biolegend
Anti-mouse IgD	11-26c.2a	BV710	Biolegend
Anti-mouse IgM	II/41	APC	BD
Anti-mouse IgG1	RMG1-1	BV421	Biolegend
Anti-mouse IgG2a	RMG2a-62	FITC	Biolegend

NP-PE		PE	Biosearch Technologies, Novato, CA, USA
**Immunohistochemistry**			
Anti-mouse B220	RA3-6B2	AF594	Biolegend
Anti-mouse GL7	GL7	BV421	Biolegend
Anti-mouse CD4	RM4-5	APC	BD

Supplementary table 2

Primers used for qRT-PCR

Target	Product code	Supplier
Igg1	Mm01742100_s1	Life Technologies, Carlsbad, CA, USA
Bcl6	Mm00477633_m1	Life Technologies
Il4	Mm00445259_m1	Life Technologies
Il21	Mm00517640_m1	Life Technologies
Cd40l	Mm00441911_m1	Life Technologies
